# Continuous flow room temperature reductive aqueous homo-coupling of aryl halides using supported Pd catalysts

**DOI:** 10.1038/srep32719

**Published:** 2016-09-07

**Authors:** Afsaneh Feiz, Ayoob Bazgir, Alina M. Balu, Rafael Luque

**Affiliations:** 1 Organic Chemistry Department, Shahid Beheshti University, Tehran 1983963113, Iran; 2 Departamento de Quimica Organica, Facultad de Ciencias, Universidad de Cordoba, Campus de Rabanales, Edificio Mariec Curie (C-3), Ctra Nnal Madrid-Cadiz, Km 396, E14014,Cordoba, Spain

## Abstract

A convenient and environmentally friendly protocol for the preparation of biaryls at room temperature under continuous flow conditions is reported. A simple reductive homo-coupling Ullmann-type reaction was performed in an H-Cube mini using commercially available supported Pd catalysts under mild reaction conditions, with quantitative conversion to target products. Commercial Pd catalysts were found to be highly stable under the investigated reaction conditions, with a minimum Pd leaching into solution after several reaction runs (ca. 20 h on stream).

Environmental concerns and the drive towards the design of greener protocols for chemicals, materials and fuels production has prompted scientists to seek alternatives to traditional high temperature/pressure, wasteful and energy intensive processes and protocols that employ or generate hazardous, explosive or toxic compounds, particularly in Organic Chemistry/Synthesis^1^,^2^,^3^. The design of environmentally sound methods and technologies is consequently an actual duty that needs to be passed to future generations aiming to a more sustainable society.

Continuous flow chemistry can offer several of the aforementioned benefits in organic chemistry and particularly for catalytic reactions^4^,^5^. These include a more careful control of reactions and processes (i.e. reaction mechanisms), more convenient scaling up to industrial scale (often impossible via conventional batch techniques), safer reactions without any need of intermediates isolation and/or generation of toxic compounds, more mass and heat transfer compared to classical batch reactors and fast mixing of materials which generally leads to very short contact and controllable reaction times (residence times)^6^. Additionally, the possibility to conduct heterogeneously catalyzed reactions under continuous flow conditions (i.e. packed bed reactors) is also a very attractive feature to potentially translate these into industrial chemical processes^6^.

Transition metal catalyzed C-C coupling reactions are one the best tools for the construction of aryl- aryl bonds present in commercial dyes, natural products, pharmaceutical compounds as well as the backbone of some ligands for catalysts^7^. A traditional route to prepare biaryl compounds, different from the typical Suzuki reactions, is the Ullmann reaction involving the homo-coupling of aryl halides^8^. This reaction was traditionally catalyzed by stoichiometric quantities of Cu, with a number of more recently literature reports disclosing the homo-coupling of aryl halides catalyzed under homogeneous or heterogeneous conditions using a range of transition metal catalysts^9^,^10^,^11^,^12^,^13^. However, most reported work involved long reaction times^14^ and harsh reaction conditions including high temperatures^15^, the use of toxic solvents^16^, strong bases^17^ and phase transfer catalysts^18^ as well as additional reducing reagents^19^,^20^. Traditionally, this reaction has been carried out in the presence of copper and/or Pd catalysts^21^ and a reducing agent (e.g. glucose, hydroquinone, sodium formate) or an oxidant as pre-requisite due to the involvement of two potential reaction pathways involving reductive coupling (Pd^2+^ species generated in the process that are reduced to Pd^0^ using an external reducing agent) and oxidative coupling (which starts with Pd^2+^ and needs an oxidizing agent)^22^,^23^.

The combination of C-C coupling processes and catalysts with continuous flow processes has not been particularly successful due to various concerns mostly related to metal catalyst stability (e.g. leaching) under flow conditions combined with temperatures and pressures^24^,^25^. A recent overview of the topic by Cantillo and Kappe pointed out the low practicality of coupling processes under continuous flow related to a generally observed metal leaching into solution based on the (quasi)homogeneous nature of coupling protocols involving the transformation of Pd^0^ into (soluble) Pd^2+^ species^25^. As compared to a batch process in which Pd^0^ typically redeposits onto the support after completion of the catalytic cycle, the metal will be gradually dissolving/redepositing through the catalyst packed bed and eventually washed out by the solvent with time on stream^25^.

Based on these premises, the development of a robust, reliable and simple setup to be able to efficiently conduct continuous flow C-C coupling reactions with a highly stable catalyst under mild reaction conditions can be extremely attractive. Previous studies of the group encompassed hydroconversion processes simply and efficiently performed in the H-Cube mini system from ThalesNano which allows a safe hydrogen gas generation in a close system from water electrolysis. We envisaged a simple application of such system to reductive homocoupling chemistries under mild reaction conditions (typically room temperature) using previously reported active Pd/C catalysts^22^,^23^. This contribution discloses one of the first examples of a room temperature continuous flow reductive Ullmann-type of homocoupling of aryl halides.

## Experimental

### General

**Figure 1:**
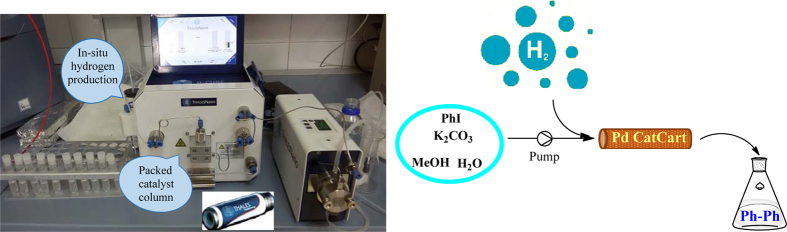
Schematic depiction of the H-Cube mini and continuous flow set up for a reductive homocoupling reaction of phenyl iodide.

All reagents and solvents were obtained from standard vendors and used without any further purification. Phenyl iodide and potassium carbonate were purchased from Sigma-Aldrich. All solutions were prepared by deionized water and ultra-pure methanol. Flow reactions were carried out in a ThalesNano H-Cube mini (Fig. 1) featuring an HPLC pump to deliver the substrates at different flow rates (typically 0.1–3 mL min^−1^ selected for our experiments), the reactor box for CatCarts (only commercial Pd catalysts were employed in this study as a proof of concept) which can be heated until 100 °C and a back pressure regulator to pressurize the system to a maximum of 100 bar. Samples were extracted after reaction and injected for analysis in an Agilent 6890N Gas Chromatograph fitted with a capillary column HP-5 (30 m × 0.32 mm × 0.25 m) and a flame ionization detector (FID). The products identity was confirmed by GC-MS.

### Ullmann reaction in continuous flow reactor

In a typical experiment for the reductive homo-coupling of aryl halides in the H-Cube mini, a solution containing 0.1 M phenyl iodide and 0.25 M potassium carbonate in a mixture of methanol:water (3:1) was pumped through a 30 mm fresh CatCart packed with different Pd catalysts (5% Pd/C and 10% Pd/C, commercially available) heated and pressurized according to the information detailed in Table 1 at a flow rate 0.3 mL min^−1^ (optimized flow conditions). With a dead volume for filled CatCarts around 208 μL, a flow rate of 0.3 mL min^−1^ means a residence time of the feed in the catalyst of ca. 42 seconds and a productivity around 2 mmol h^−1^.

The solution was passed through the catalyst for 2 h (20 min reaction is equivalent to the aforementioned 42 s residence time), with samples being taken every 5–10 mins for GC analysis. Investigated Pd catalysts included 5 and 10% Pd/C commercial materials from Alfa Aesar and 10% Pd-PBSAC kindly provided by SARATECH Blucher technologies. 5% Pd/C contained 4.93 wt% Pd (metal area of 20 m^2^ g^−1^) while 10% Pd/C contained a 9.87 wt% Pd (18 m^2^ g^−1^ metal are 9–12. For 10% Pd-PBSAC, where PBSAC stands for SATARECH Polymer based Spherical Activated Carbon, the material were monodispersed spheres with ca. 75 μm average diameter, >95% carbon content) in which Pd nanoparticles with average Pd particle sizes <5 nm were supported by simple impregnation. Control experiments in the absence of hydrogen were also made with non hydrogen donating solvents (e.g. acetone) for which essentially identical results to those with methanol were obtained (no hydrogen transfer occurs at the investigated reaction conditions).

Investigations on Pd leaching were conducted on several randomly selected samples (both from the final reaction mixture after 2 h and 20 h as well as samples of washings with solvents after every catalyst reuse) using a Philips PU 70000 sequential spectrometer equipped with an Echelle monochromator (0.0075 nm resolution) and coupled to mass spectrometry (ICP-MS). Collected samples (1 mL) were digested in HF:HNO_3_:HCl and subsequently analyzed at the SCAI of Universidad de Cordoba via ICP-MS.

## Results and Discussion

The reductive homo-coupling of aryl halides using different transition metal catalysts has been reported in the literature^26^,^27^. Bimetallic gold/palladium alloy nanoclusters were employed in the homocoupling of aryl chlorides^17^ as well as gold NPs stabilized on nanocrystalline magnesium oxide for aryl iodides in DMF^15^. From most employed Pd catalysts, supported palladium NPs have also been utilized for Heck and Ullmann couplings in the absence of any reducing agent mediated by a phase transfer catalyst^28^ in a similar way to palladium NPs on graphene catalyzing Suzuki and Ullmann couplings^7^.

As a model optimization reaction, we initially conducted the homo-coupling of phenyl iodide using commercial 10% Pd catalysts (both 10% Pd/C and 10% Pd/PBSAC) in a methanolic aqueous mixture. A 3:1 methanol: water ratio was utilized as optimum due to the poor solubility of the reaction mixture in neat water or a 1:1 mixture. Initial reaction conditions included 90 °C temperature, 8 bar hydrogen pressure and 0.3 mL min^−1^ flow rate (optimum) of starting materials (Fig. 1), in order to mimic typical reaction conditions for batch coupling catalyzed reactions^25^. Higher flow rates (>0.3 mL min^−1^) were not considered as a significant Pd leaching in the systems was observed under such conditions.

**Figure 2:**
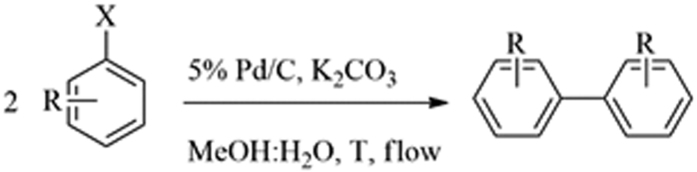
Pd-catalysed Ullmann coupling at room temperature.

Blank runs (in the absence of catalyst, Cat Cart filled with quartz sand) did not provide any conversion of starting materials in the system. Comparably, reaction runs with both 10% Pd/C and 10% Pd/PBSAC as catalyst provided quantitative conversion to biphenyl after only about 40 seconds of residence time. A decrease in Pd loading to 5% Pd/C did not seem to have any effect in the systems, with quantitative conversion to biphenyl after the same reaction time. An optimization of reaction temperatures, hydrogen pressures and Pd loading in the catalyst was then conducted to find out the mildest possible reaction conditions at which the proposed reductive homocoupling could be performed. The proposed Ullmann coupling (Fig. 2) was highly selective, with biphenyl exclusively obtained as reaction product. No hydrodehalogenation compounds or related byproduct could be observed (even at temperatures as high as 90 °C), most probably due to the mild conditions employed.

To our delight, the homocoupling of both phenyl iodide and phenyl bromide could be efficiently conducted at room temperature and a hydrogen pressure as low as 2 bar (Table 1, entries 11 and 12) after ca. 42 seconds of residence time, 20 mins on stream, which seemed to be critical for the reaction to take place. Performing the reaction in the absence of hydrogen or catalyst provided virtually no conversion in the systems, indicating the requirement of the reducing agent (hydrogen) and a metal-based catalyst (Pd) for the reaction to take place. Reactions performed with similar acetone:water mixtures (in the absence of methanol) provided essentially identical results in the coupling reactions, ruling out the involvement of hydrogen transfer in the methanol:water systems (in any case unlikely to be the case due to the room temperature protocol).

**Figure 3:**
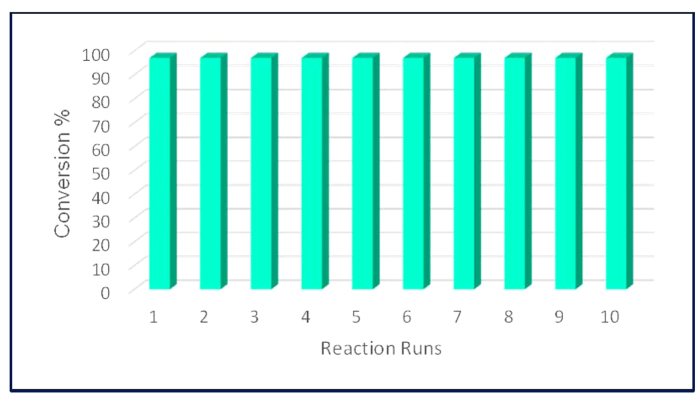
Investigation of the catalysis performance in 10 runs. (Reaction conditions: 0.1 M PhI and 0.25 M K_2_CO_3_ in MeOH:H_2_O 3:1, 0.3 mL.min^−1^, 5% Pd/C; 2 h each reaction run).

In principle, no presence of leached Pd black could be observed into solution after reaction, pointing to a good stability of both Pd/C (5 and 10%) and Pd/PBSAC catalysts. Nevertheless, a detailed study for Pd leaching and catalyst characterization in the systems as well as the presence of other metals as potential reaction catalysts was subsequently conducted. A number of collected samples were firstly analysed by ICP-MS. These included a) the final mixture after reaction (2 h on stream, ca. 250 s residence time); b) the final mixture after a long reaction run (20 h continuous run); the final mixture after 10 reuses of the catalyst (20 h discontinuous run) as well as solvent washings from catalyst conditioning for 5% Pd/C after each run. Results indicated an almost negligible leaching (maximum ca. 10 ppm, generally <5 ppm) of Pd into solution in all cases under optimized reaction conditions, with the exception of the final mixture after 20 h of continuous run (cumulative solution), in which a relevant Pd concentration was observed (>100 ppm). A careful investigation of the Pd content in 5% Pd/C before and after reaction (as well as in the washings) further confirmed the negligible leaching quantified by ICP-MS in most cases, with the exception of the long reaction run experiment. In this case, a decrease of Pd content in the catalyst was observed (a reduction from 4.93 wt.% Pd in the fresh catalyst to 4.49 wt.% Pd in the catalyst after the long reaction run). Despite the observed quantified leaching in long run experiments, these findings pointed out a generally good stability of the catalytic system at low flow rates and room temperature conditions. Comparably, the reuses of Pd catalysts (5% Pd/C, each one of them after 2 h reaction) under room temperature optimized reaction condition were proved to be highly successful even after ten consecutive reaction runs (accounting for a total of 20 hours in flow) without any significant loss of activity and/or selectivity (Fig. 3).

The optimized room temperature continuous flow protocol was also amenable to a range of aryl halides including bromoarenes (bromobenzene and 4-bromoanisole) and more challenging chloroarenes (4-chlorobenzaldehyde, Table 1, entry 14). In all cases, a complete selectivity to the biphenyl derivative was also obtained, with no major side products obtained for any of the proposed reactions under the optimized reaction conditions. Interestingly, the carbonyl groups from the homocoupling product from 4-chlorobenzaldehyde ([1,1-byphenyl]-4,4′-dicarbaldehyde, Table 1, entry 14) were gradually hydrogenated with time on stream to the corresponding hydroxyls even at room temperature and low hydrogen pressures to an almost quantitative yield to [1,1-byphenyl]-4,4′-diyldimethanol after 2 h of stream (252 s residence time). These unexpected findings pointed out the possibility to conduct multistep one-pot catalytic reactions at room temperature (e.g. couplings-hydroconversion) that will be further explored in due course.

**Figure 4:**
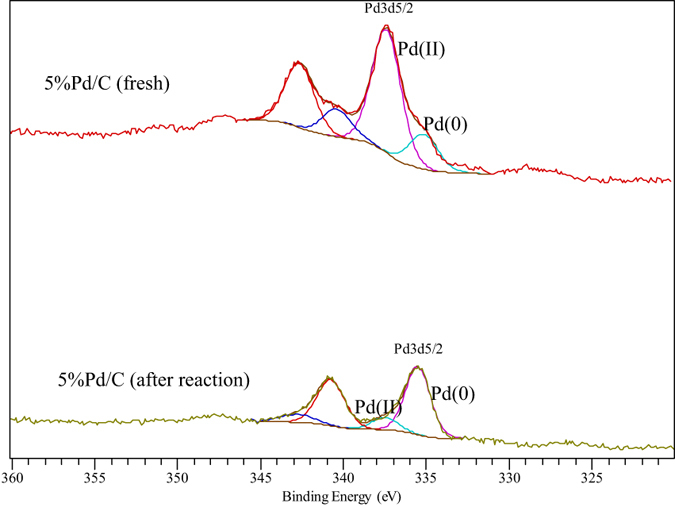
XPS spectra of 5% Pd/C catalyst before and after the room temperature continuous flow Ullmann reaction. Reaction conditions (for the 5% Pd/C after reaction: 0.1 M PhI and 0.25 M K_2_CO_3_ in MeOH:H_2_O 3:1, 0.3 mL.min^−1^, 2 h on stream).

To the best of our knowledge, these are probably the best results reported to date in terms of catalyst stability for a heterogeneous Pd system in continuous flow C-C couplings at room temperature in aqueous conditions^22^,^23^,^25^,^29^. Characterisation of the 5% Pd/C by XPS (Fig. 4) before and after reaction provided some interesting insights into a plausible reaction mechanism and the observed relatively good stability of the systems. Figure 4 shows a typical batch of the commercial 5% Pd/C catalyst features Pd(II) species as major contribution present in the materials at 337.5 eV, with a minor proportion (<20%, see deconvoluted contribution at 334.8 eV) of Pd(0) nanoparticles. Interestingly, XPS spectra of 5% Pd/C after reaction (2 h on stream) already shows almost completely reduced Pd(0) species, with a very minor proportion of Pd(II), see Fig. 4 bottom, in good agreement with recently reported findings of the group on flow hydrogenation reactions^30^.

**Figure 5:**
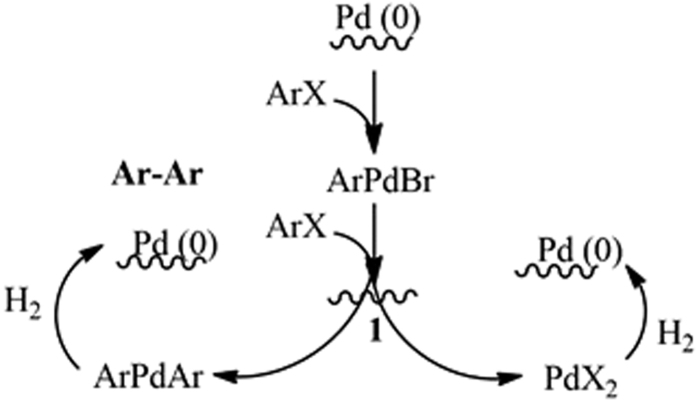
Proposed mechanism for the Pd-catalysed Ullmann reaction.

The reaction mechanism has been previously reported in literature for a number of Pd-catalysed Ullmann reactions^21^,^22^,^23^, being still unclear. Based on the presence of a majority of Pd(II) species in the initial catalyst, a simple proposed mechanism will initially involve the coordination of two aryl halide molecules to Pd(II) species (**1**) to generate Ar-Pd-Ar intermediates and PdX_2_ species. Ar-Pd-Ar intermediates then underwent reductive elimination towards Ar-Ar (biaryl) products in which the hydrogen provided in the continuous flow system will generate Pd(0) species, in the same way that PdX_2_ may also react with H_2_ to generate Pd(0) species as observed in increasing quantities with time on stream. We believe the initial presence of Pd(II) species in the catalyst may contribute to the excelling rates of reaction observed at room temperature under continuous flow conditions (no induction period observed in terms of reactivity), with hydrogen being essential to close the catalytic cycle, particularly for the proposed reductive elimination step and the formation of Pd(0) species able to continue the cycle as illustrated in Fig. 5.

## Conclusions

A very short, simple, efficient and aqueous room temperature reductive homo-coupling of aryl halides was successfully carried out in quantitative yields under continuous flow conditions using commercial available Pd catalysts. The catalytic systems were found to be relatively stable under the investigated reaction conditions, without any significant Pd leaching (as quantified by ICP-MS) only evidenced for long reaction runs (20 hours continuously). Harsh reaction conditions including high temperature, long reaction times and utilization of an excess of reducing agents were replaced with very mild conditions (room temperature) and short times of reaction (ca. 42 seconds). We envisaged these types of chemistries to be further extended to a range of designed active and stable transition-metal catalysts that will be reported in due course.

## Additional Information

**How to cite this article**: Feiz, A. *et al*. Continuous flow room temperature reductive aqueous homo-coupling of aryl halides using supported Pd catalysts. *Sci. Rep.* **6**, 32719; doi: 10.1038/srep32719 (2016).

## Figures and Tables

**Table 1:** Continuous flow Ullmann reaction catalyzed by 5% Pd/C in the H-Cube mini.

Entry	Temperature (°C)	Pressure (bar)	Conversion (%)
1^a^	90	8	—
2	90	8	>99
3	90	5	>99
4	80	5	>99
5	80	2	>99
6	60	2	>99
7	40	2	>97
8	40	H_2_ Off	<10
9^b^	40	2	>99
10^c^	40	2	>97
11	25	2	>97
12^c^	25	2	>98
13^d^	25	2	>98
14^e^	25	2	>90

Reaction conditions: 0.1 M PhI and 0.25 M K_2_CO_3_ in MeOH: H_2_O 3:1, 0.3 ml.min^−1^, 5% Pd/C, residence time: 42 seconds residence time, 20 min on stream;

^a^Blank reaction with inert CatCart.

^b^0.5 mL.min^−1^ flow rate.

^c^0.1 M PhBr, 0.3 mL.min^−1^.

^d^0.1 M 4-bromoanisole, 0.3 mL.min^−1^.

^e^0.1 M 4-chlorobenzaldehyde, 0.3 mL.min^−1^.
